# Cytologic and Histological Features Distinguishing Myxoid Meningioma From Chordoid Meningioma: A Case Report

**DOI:** 10.1002/dc.70088

**Published:** 2026-01-27

**Authors:** Tatsuya Aso, Miyuki Yoshino, Manabu Fukuda, Kazuhiko Yuzawa, Mikiko Takahashi

**Affiliations:** ^1^ Department of Diagnostic Pathology University Hospital, Mizonokuchi, Teikyo University School of Medicine Kawasaki City Kanagawa Japan

**Keywords:** chordoid meningioma, metaplastic meningioma, myxoid meningioma, WHO grade

## Abstract

Meningiomas are the most common primary brain tumors worldwide and are classified into 15 subtypes in the 5th edition of the WHO classification. Myxoid meningioma, characterized by the presence of a mucinous matrix within the tumor, is a rare metaplastic meningioma subtype classified as WHO grade 1. Chordoid meningiomas similarly contain a mucinous matrix, but are WHO grade 2. Accurate distinction between these subtypes is essential for determining appropriate treatment and predicting prognosis. Herein, we present a case of myxoid meningioma requiring differential diagnosis from chordoid meningioma. A man in his 60s presented with falls, depression, and urinary incontinence. Imaging revealed a tumor 6 cm in diameter in the right frontal region. Tumor resection with rapid intraoperative diagnosis was performed. Cytology revealed epithelioid cells with oval nuclei, intranuclear cytoplasmic inclusions, and mucinous matrix. Squash cytology confirmed tumor cell clusters with a vascular network. Histological examination revealed tumor cells forming cord‐like structures within an Alcian blue‐positive, Periodic acid–Schiff‐negative mucinous matrix, along with an abundant vascular architecture. Myxoid and chordoid meningiomas share many cytological similarities, and mucin staining patterns are diagnostically unclear. However, the latter tend to show lymphocytic and plasma cell infiltration. This case lacked such infiltration, and the vascular stroma aided differentiation. For rapid intraoperative diagnosis, a combination of frozen sections and cytology, less susceptible to freezing artifacts, is considered beneficial for accurate diagnosis. In meningiomas with a mucinous matrix, careful evaluation of cellular appearance and tumor stroma findings is essential for distinguishing between subtypes with different WHO grades.

## 1 Introduction

Meningiomas are the most common type of primary brain tumors worldwide [1]. The 5th edition of the World Health Organization (WHO) classifies meningiomas into 15 subtypes, for which treatment approaches and recurrence risk are dependent on the WHO grade, an indicator of malignancy [1]. Among these, metaplastic meningiomas are a rare subtype, and myxoid meningioma, a variant of metaplastic meningioma, is classified as WHO grade 1 [1]. Chordoid meningioma, which also produces a mucinous matrix similar to that of myxoid meningioma, is classified as WHO grade 2, indicating a higher malignancy grade [1]. Distinguishing between these two subtypes is clinically important. Myxoid meningioma is a rare metaplastic subtype with very few reported cases, and its clinicopathological and cytological features are poorly defined [2, 3]. Histologically, it comprises spindle to oval tumor cells arranged in cords or small nests within an abundant myxoid (mucoid) stroma. The amount of myxoid matrix varies among cases, and no quantitative threshold for diagnosis has been established. The tumor cells show bland, elongated nuclei with inconspicuous nucleoli and eosinophilic cytoplasm; small vessels may be present within the myxoid stroma [2, 3]. Reports describing the cytological features of myxoid meningioma are extremely scarce, making intraoperative cytological diagnosis challenging. Based on limited descriptions, cytologic smears may show loosely cohesive cords of uniform spindle cells in a homogeneous, Alcian blue–positive background, usually lacking inflammatory components [2, 3]. In contrast, chordoid meningioma has been more frequently characterized cytologically. Smears typically demonstrate polygonal to epithelioid tumor cells arranged in cords or clusters within a mucinous, PAS‐positive background [3, 4]. The cytoplasm tends to be more abundant and sometimes vacuolated, with round nuclei and occasional intranuclear cytoplasmic inclusions. A distinguishing feature of chordoid meningioma is the presence of lymphoplasmacytic infiltration in the background, an element that is rarely observed in myxoid meningioma [3, 4, 5, 6]. Because of their overlapping mucinous morphology, differentiating myxoid meningioma (WHO grade 1) from chordoid meningioma (WHO grade 2) can be challenging on cytology alone. Nevertheless, recognizing subtle cytological differences—such as cell shape, cytoplasmic character, and the presence or absence of inflammatory cells—can aid accurate intraoperative interpretation. Herein, we discuss the cellular features of both subtypes and the key points for distinguishing them based on their surrounding tissues.

## 2 Case Presentation

**FIGURE 1:**
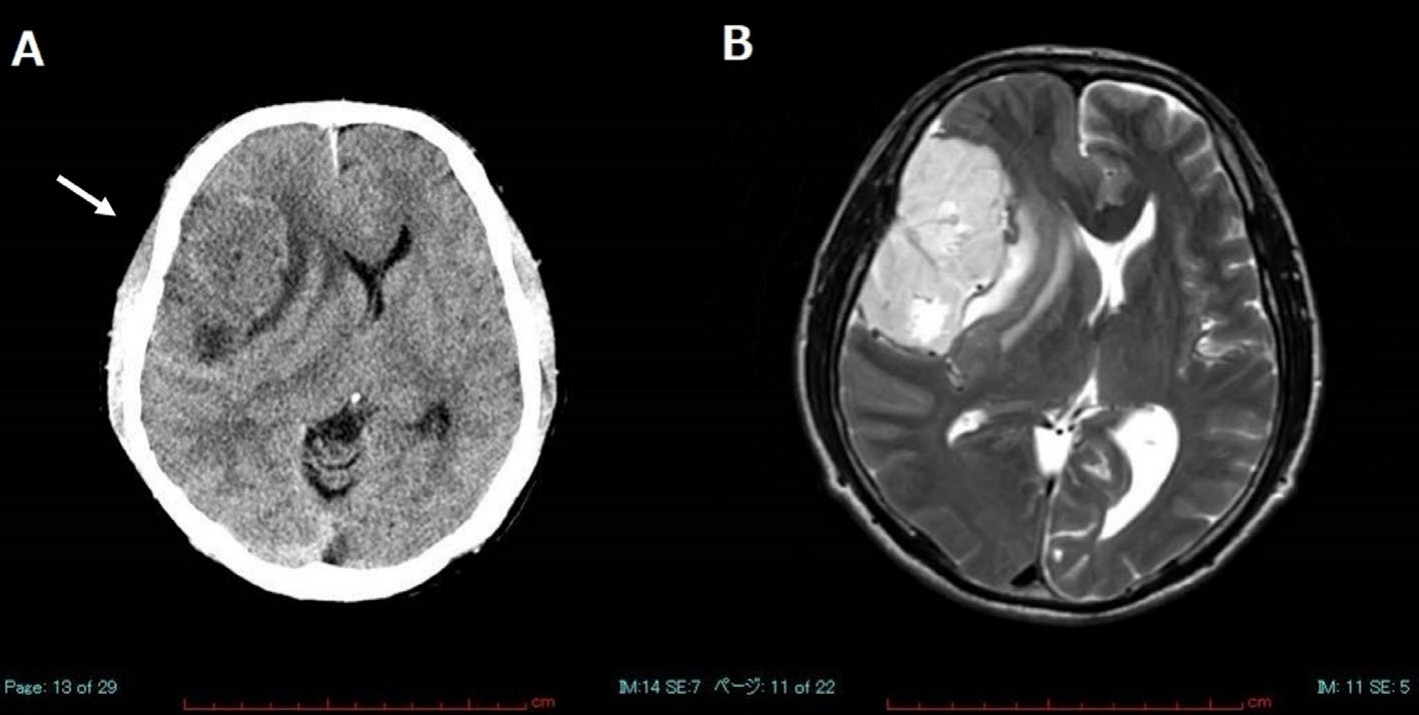
(A) Computed tomography showing an irregularly shaped, hypodense tumor (arrow) measuring approximately 6 cm in diameter in the right frontal dome extending to the sphenoid margin. (B) T2‐weighted magnetic resonance imaging showing high signal intensity.

A 60‐year‐old man who had begun experiencing frequent falls and developed symptoms, including depression and urinary incontinence, approximately 1 year prior, visited our hospital. Head computed tomography (CT) revealed an irregularly shaped tumor measuring approximately 6 cm in diameter, extending from the right frontal dome to the sphenoid margin. Head magnetic resonance imaging (MRI) revealed a high signal intensity of the lesion on T2‐weighted imaging (Figure 1A,B). The lesion appeared to be an extra‐axial tumor with broad dural attachment and a dural tail sign, suggestive of a meningioma or solitary fibrous tumor. Subsequently, tumor resection with rapid intraoperative diagnosis was performed.

**FIGURE 2:**
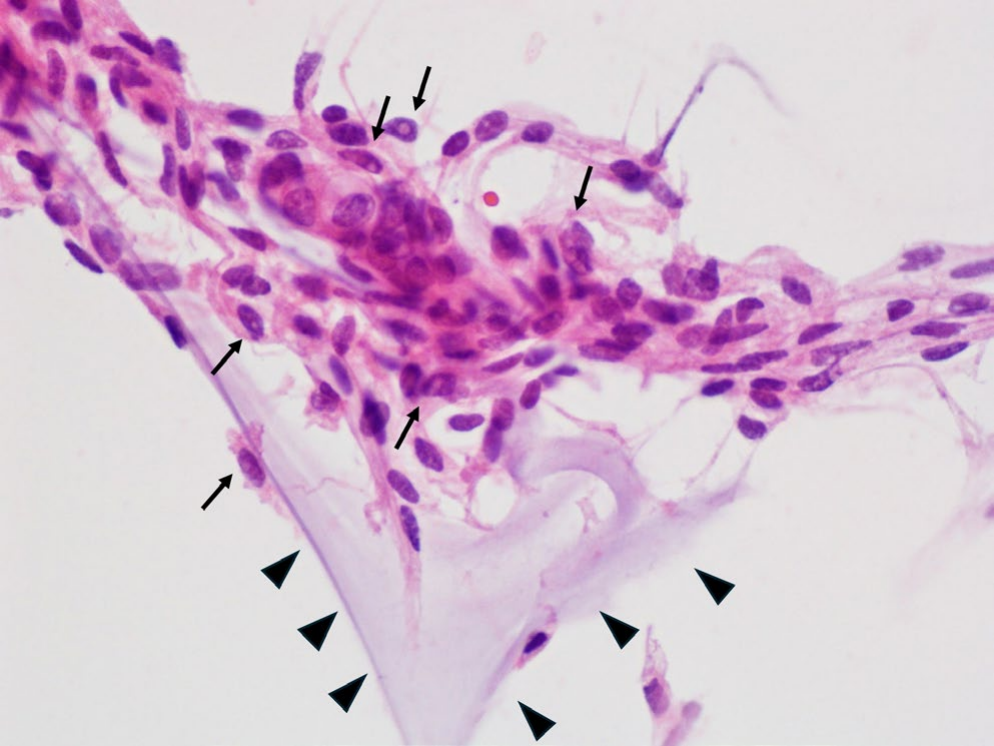
Hematoxylin and eosin staining (squash cytology, ×60) showing tumor cells with intranuclear cytoplasmic inclusions (arrow) and a mucus‐like substance stained by hematoxylin (arrowhead).

**FIGURE 3:**
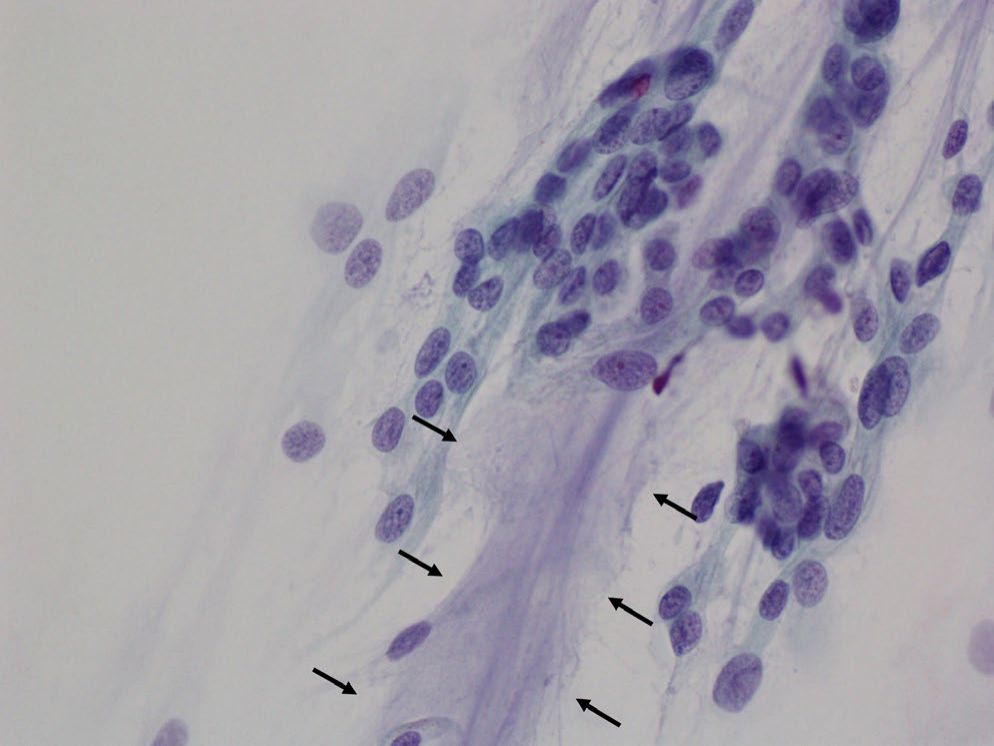
Stamp cytology (Papanicolaou stain, ×60) showing epithelioid cells with variable nuclear size and pale green–stained fibrous cytoplasm; the mucinous matrix is stained with hematoxylin (arrow).

**FIGURE 4:**
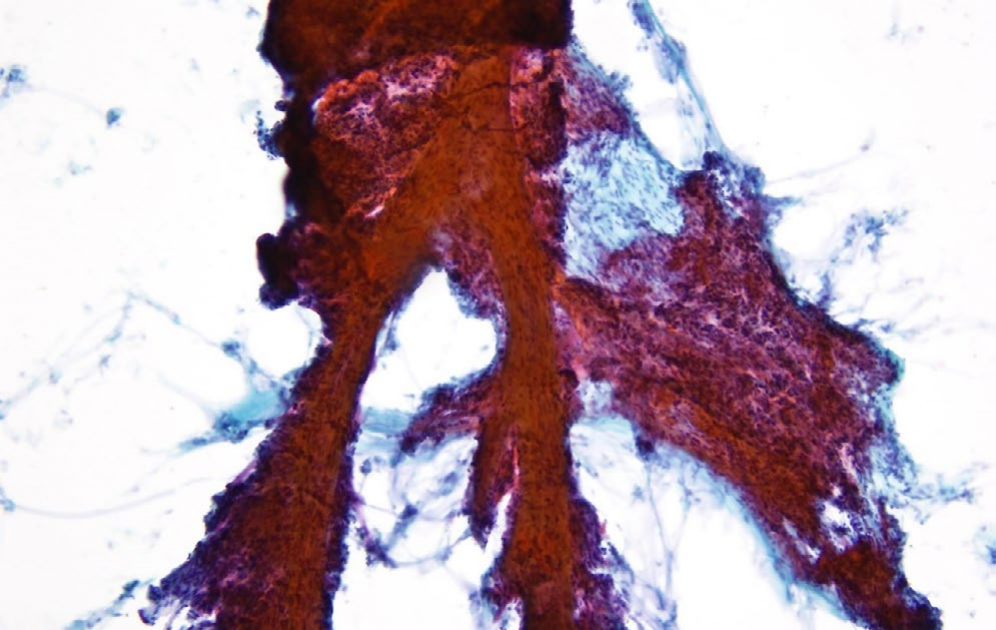
Squash cytology (Papanicolaou stain, ×10) showing tumor cell clusters containing relatively thick, branching blood vessels.

The cytological analysis revealed epithelioid cells with oval nuclei. Some cells contained intranuclear cytoplasmic inclusions and a hematoxylin‐positive mucinous matrix (Figures 2, 3). No lymphocytic or plasmacytic infiltration was observed in the background. Squash cytology further demonstrated branching blood vessels running through the center of the tumor cell clusters (Figure 4).

**FIGURE 5:**
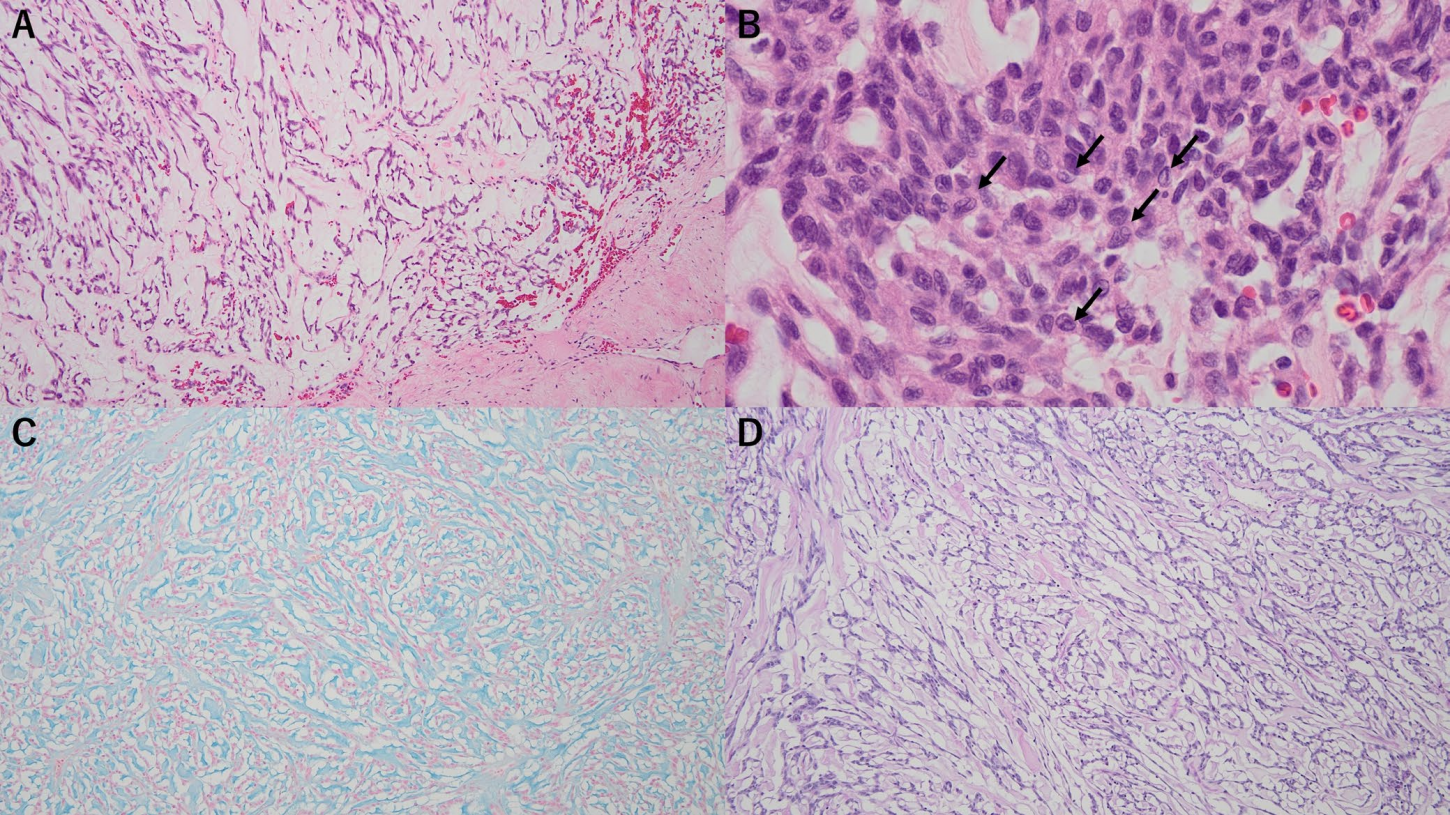
(A) Tumor cells proliferating and adhering to the dura mater within an abundant mucinous matrix (H&E, ×10). (B) Tumor cells showing intranuclear cytoplasmic inclusions (H&E, ×60) (arrow). (C) Alcian blue staining (pH 2.5, ×10). (D) Periodic acid–Schiff staining (×10), showing Alcian blue–positive and PAS‐negative mucinous matrix.

The histological findings were as follows: The tumor adhered to the dura mater and formed cord‐like structures within a mucinous matrix containing numerous vessels of varying sizes. Occasional tumor cells exhibited intranuclear cytoplasmic inclusions, corresponding to the findings observed in the cytological specimens and further supporting meningothelial differentiation (Figure 5A,B). The mucinous matrix was Alcian blue positive and Periodic acid–Schiff negative (Figure 5C,D). No lymphocytic or plasmacytic infiltration was present within or around the tumor, and psammoma bodies were not identified. Mitotic figures were rare, and necrosis was not observed. Furthermore, the Ki‐67 labeling index was 2%. Immunohistochemically, the tumor was negative for cytokeratin (AE1/AE3), and positive for epithelial membrane antigen (EMA), vimentin, and progesterone receptor (PgR). The tumor exhibited an abundant myxoid stroma but showed morphological and immunophenotypic features characteristic of meningioma, with no evidence of mitosis or necrosis; therefore, it was diagnosed as a myxoid meningioma. Approximately 7 years post‐surgery, the patient has not experienced recurrence.

## 3 Discussion

Metaplastic meningioma is characterized by the presence of bone, cartilage, adipose tissue, myxoid components, and xanthoma‐like components, either alone or in combination. These features are relatively uncommon in meningiomas [7]. The constituent elements of metaplastic meningioma are often predominantly bone or adipose tissue. Myxoid meningiomas, which are primarily composed of myxoid components, are even rarer, with fewer reported cases [8]. Distinguishing myxoid meningiomas from chordoid meningiomas, which produce a similar mucinous matrix, within the meningioma spectrum is crucial. Myxoid meningioma is classified as WHO grade 1, whereas chordoid meningioma is classified as WHO grade 2, leading to differences in treatment approaches and prognosis [1]. Both share common features; tumor cells exhibit short spindles or round nuclei, and intranuclear cytoplasmic inclusions may be observed. Cytoplasmic vacuolar degeneration has also been reported in both subtypes [9, 10, 11, 12, 13, 14]. Regarding mucus characteristics, staining patterns with alcian blue and Periodic acid–Schiff stain vary, while no specific findings have been reported as potential diagnostic markers [9, 10, 11, 14]. Noncellular features include a vascular network in myxoid meningiomas and lymphocytic and plasma cell infiltration in chordoid meningioma [9, 10, 13, 14]. In the present case, the diagnosis of myxoid meningioma was established based on morphological, immunohistochemical, and exclusionary findings. The tumor demonstrated cords and nests of spindle to oval cells within a myxoid stroma rich in small vessels. Typical meningothelial whorls were inconspicuous, although focal concentric cell clustering supported meningothelial differentiation. No mitoses or necrosis were observed. Immunohistochemically, the tumor cells were positive for EMA, vimentin, and PgR, while cytokeratin (AE1/AE3) was negative. The main differential diagnoses included chordoid meningioma and metastatic mucinous carcinoma. Notably, there was no lymphocytic or plasmacytic infiltration within or around the tumor, a finding that supports the diagnosis of myxoid meningioma. Metastatic carcinoma was excluded based on the lack of cytokeratin expression and cytologic atypia. Rapid intraoperative diagnosis is crucial for brain tumors, including meningiomas. Depending on the patient's condition, the surgical approach may differ between subtotal resection and gross total resection, based on the WHO grade [15]. However, the amount of tissue obtained is often minimal, and the impact of freezing artifacts on frozen sections cannot be ignored. It is therefore important to combine histological diagnosis with cytology, which is unaffected by freezing, and to incorporate both the characteristics of tumor cells and findings in the surrounding tissue as elements of the diagnosis. Given the limited number of reported cases for both subtypes, further case accumulation and comparative analyses are highly anticipated.

## Funding

The authors did not receive support from any organization for the submitted work.

## Ethics Statement

Our institute does not require ethical approval for the publication of case reports.

## Consent

The patient provided written informed consent for publication of this report.

## Conflicts of Interest

The authors declare no conflicts of interest.

## Data Availability

Data sharing not applicable to this article as no datasets were generated or analysed during the current study.
